# Supplementary material for: BRCA2 controls DNA:RNA hybrid level at DSBs by mediating RNase H2 recruitment
Source: Nat Commun. 2018 Dec 18;9:5376. doi: 10.1038/s41467-018-07799-2 (PMC6299093; doi:10.1038/s41467-018-07799-2)
Supplement: Supplementary file 1 — Supplementary Information [file 41467_2018_7799_MOESM1_ESM.pdf]

Supplementary Information

BRCA2 controls DNA:RNA hybrid level at DSBs by mediating RNase H2 recruitment

Giuseppina D'Alessandro[1*], Donna Rose Whelan[2], Sean Michael Howard[3], Valerio Vitelli[1], Xavier Renaudin[4], Marek Adamowicz[1,5], Fabio Iannelli[1], Corey Winston Jones-Weinert[1], Miyoung Lee[4], Valentina Matti[1], Wei Ting C. Lee[2], Michael John Morten[2], Ashok Raraakrishnan Venkitaraman[4], Petr Cejka[3,6], Eli Rothenberg[2] and Fabrizio d'Adda di Fagagna[1,7,*]

1 IFOM, the FIRC Institute of Molecular Oncology, Via Adamello 16, 20139 Milan, Italy;
2 Department of Biochemistry and Molecular Pharmacology, NYU School of Medicine, New York, NY 10016, USA;
3 Institute for Research in Biomedicine, Università della Svizzera italiana, Via Vela 6, 6500 Bellinzona, Switzerland
4 Medical Research Council Cancer Unit, University of Cambridge, Hills Road, Cambridge CB2 0XZ, UK.
5 current address: Genome Damage and Stability Centre, School of Life Sciences, University of Sussex, Falmer, Brighton BN1 9RH, UK.
6 Department of Biology, Institute of Biochemistry, Swiss Federal Institute of Technology, Otto-Stern-Weg 3, 8093 Zurich, Switzerland
7 Istituto di Genetica Molecolare, Consiglio Nazionale delle Ricerche (IGM-CNR), Via Abbiategrasso 207, 27100 Pavia, Italy.
* Corresponding authors: fabrizio.dadda@ifom.eu, giuseppina.dalessandro@ifom.eu

a

Site	Genome	Position	Annotation	Gene	Upstream Gene	Upstream Gene Distance (bp)	Downstream Gene	Downstream Gene Distance (bp)
I-PpoI genic	Human GRCh37/hg19	chr1:58,453,282-58,453,296	Intronic	DAB1	DAB1	0	DAB1	0
I-PpoI non-genic	Human GRCh37/hg19	chr2:133,037,429-133,037,443	Intergenic	-	ANKRD30BL	21888	ZNF806	27274
AsiSI non-genic	Human GRCh37/hg19	chr20:37,360,268-37,360,276	Intergenic	-	SLC32A1	2254	ACTR5	16281

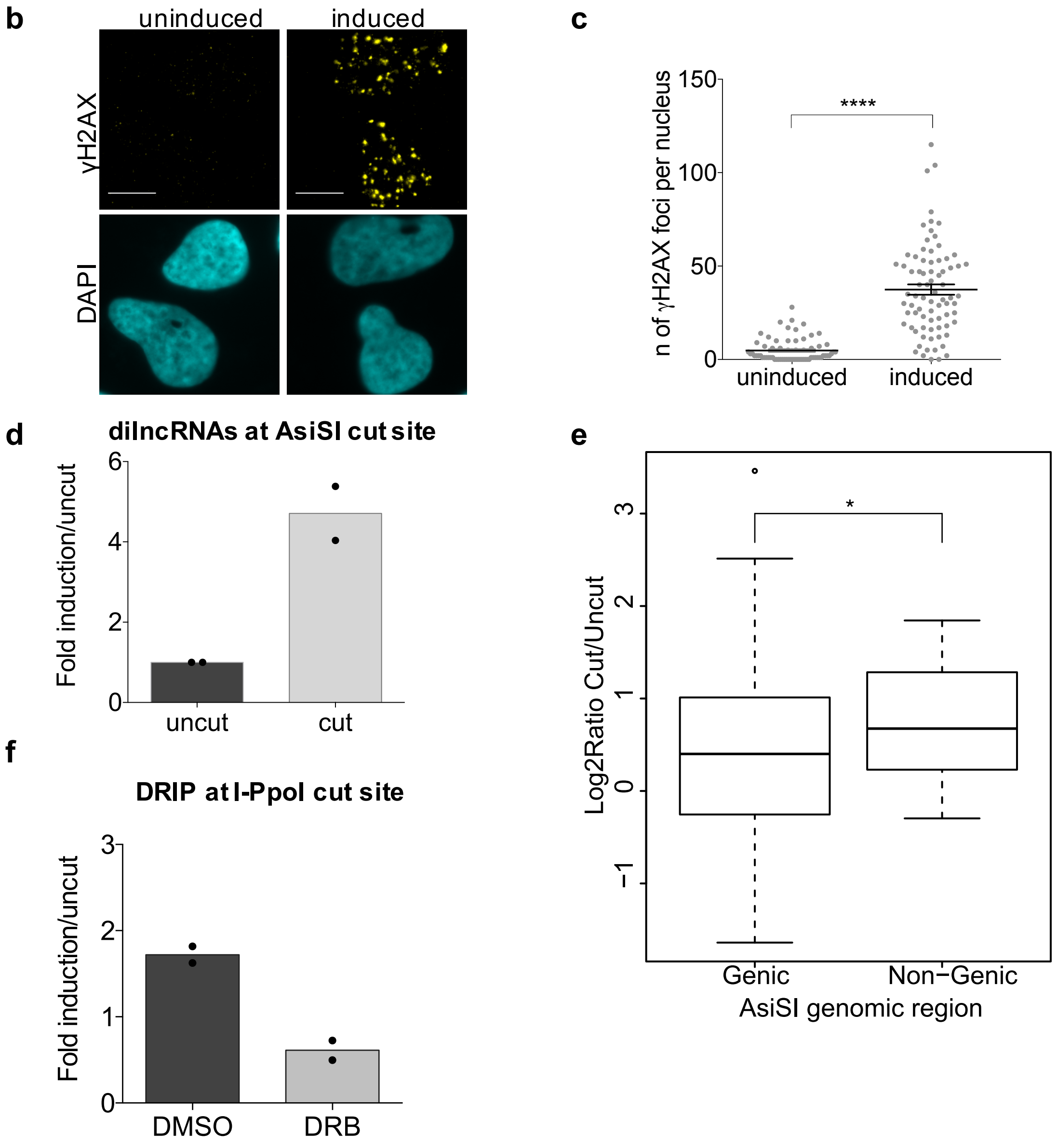

Supplementary Fig. 1: DNA:RNA hybrids formation at DSBs

a, Table indicating the coordinates and the genomic context of the restriction enzymes-induced DSBs in the study **b**, Representative images of γH2AX staining in DIvA cells before and after AsiSI induction with 4-OHT. Scale bar: 10 µm. **c**, Dot plot shows the number of foci in **b**. At least $n = 60$ cells were counted from 2 independent experiments. Lines represent mean±s.e.m. **d**, dilncRNAs induction at 0.5 Kb from the AsiSI cut site in DIvA cells measured by strand-specific RT–qPCR. The bar graph shows the average fold induction of cut samples relative to uncut from $n = 2$ independent experiments. *$P < 0.05$, ****$P < 0.0001$ (two-tailed Student's t test). **e**, Boxplot representing log2 ratio of the fold change of DNA:RNA hybrids reads in cut compared to uncut samples at the BLISS-detected top 50 genic or non-genic AsiSI sites. *$P < 0.05$ (Wilcoxon Rank Sum Test). **f**, DRIP-qPCR at 1.5 Kb on the right from the I-PpoI cut site within *DAB1* gene in HeLa cells treated with DMSO or DRB for 2 hours prior to DSB induction. The bar graph shows the average fold induction of cut samples relative to uncut from $n = 2$ independent experiments. *$P < 0.05$ (two-tailed Student's t test). Source data are provided as a Source Data file.

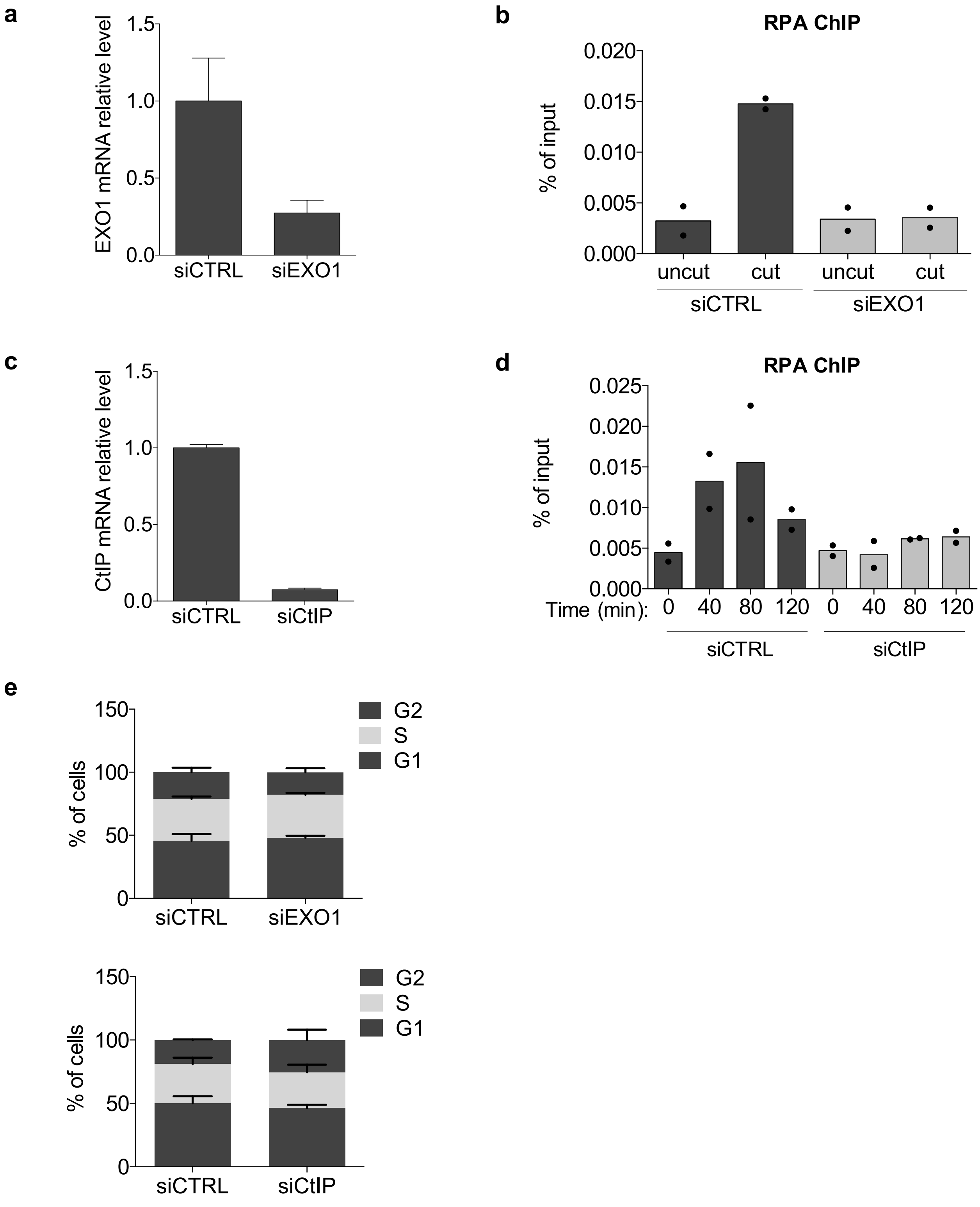

Supplementary Fig. 2: CtIP and EXO1 knock-down impair DNA-end resection

a, EXO1 knock-down efficiency monitored by RT-qPCR. One representative experiment is shown. Error bars represent s.d. of the RT-qPCR technical replicates. **b**, Accumulation of RPA at 1.5 Kb on the right from the I-PpoI cut site within *DAB1* gene in HeLa cells knocked-down for EXO1. **c**, CtIP knock-down efficiency monitored by RT-qPCR. One representative experiment is shown. Error bars represent s.d. of the RT-qPCR technical replicates. **d**, Accumulation of RPA at 1.5 Kb on the right from the I-PpoI cut site within *DAB1* gene in HeLa cells knocked-down for CtIP at different time points after cut. Bar graphs in **b** and **d** show the percentage of input of total RPA from $n = 2$ biological replicates. **e**, FACS analyses of the cell-cycle profile of cells knocked-down for CtIP or EXO1. Bar graphs represent mean values from $n = 2$ independent experiments and error bars represent s.e.m. Source data are provided as a Source Data file.

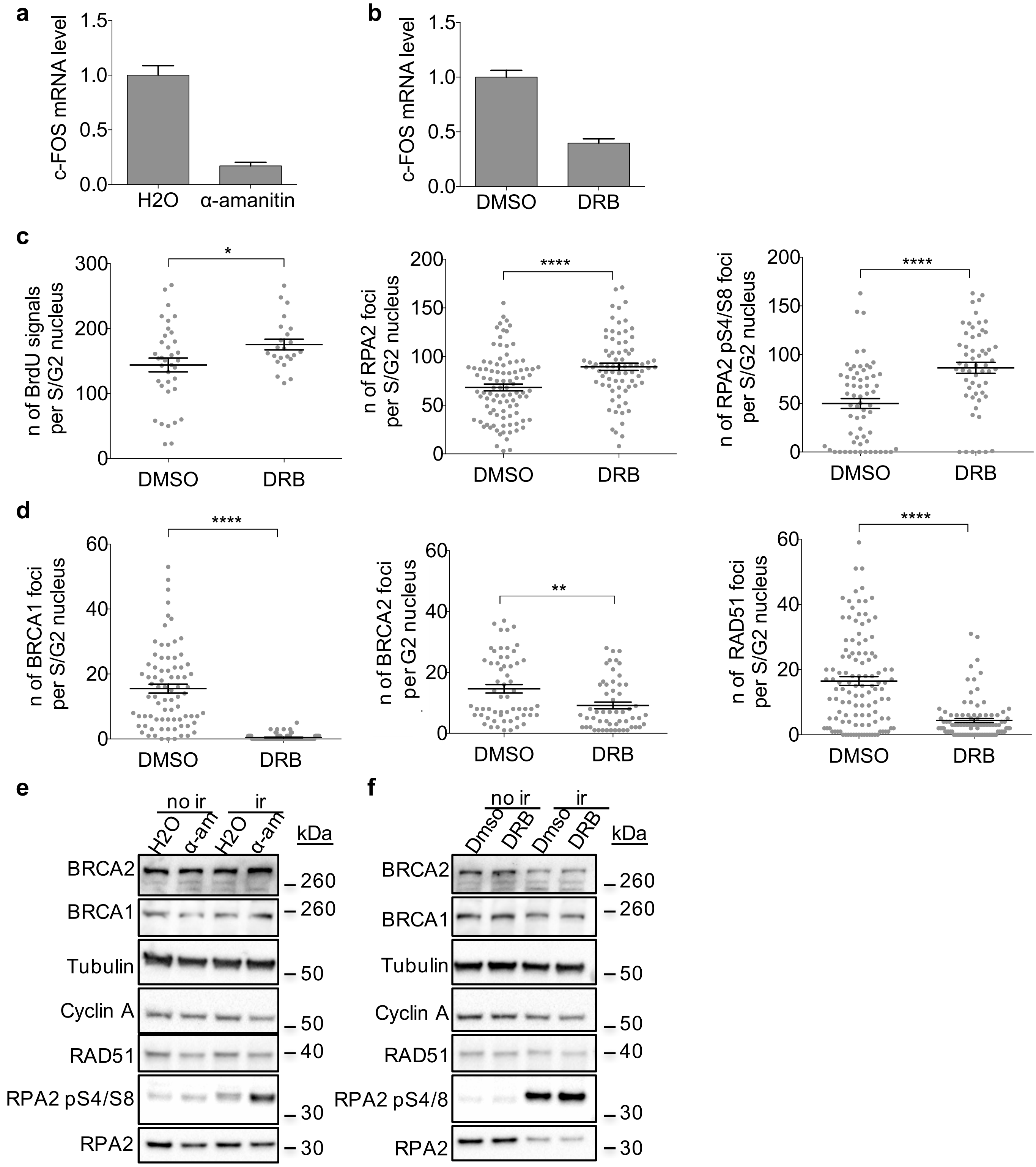

Supplementary Fig. 3: Transcriptional inhibition does not significantly alter HR protein levels

a, Efficiency of α-amanitin or **b**, DRB treatment monitored by RT-qPCR analysis of the mRNA levels of *c-FOS*, a short-lived RNA pol II transcript. One representative experiment is shown. Error bars represent s.d. of the RT-qPCR technical replicates. **c**, Dot plots show the number of signals/foci per S/G2 nucleus of the indicated DNA-end resection or **d**, HR markers. At least $n = 50$ (except for BrdU staining, $n \geq 20$) from 2 independent experiments were counted. Lines represent mean±s.e.m. **e**, Representative immunoblots of not irradiated (no ir) or irradiated 5Gy (ir) HeLa cells treated with α-amanitin or **f**, DRB or relative vehicles. These experiments were repeated twice independently with similar results. *$P < 0.05$, **$P < 0.01$, ****$P < 0.0001$ (two-tailed Student's t test). Source data are provided as a Source Data file.

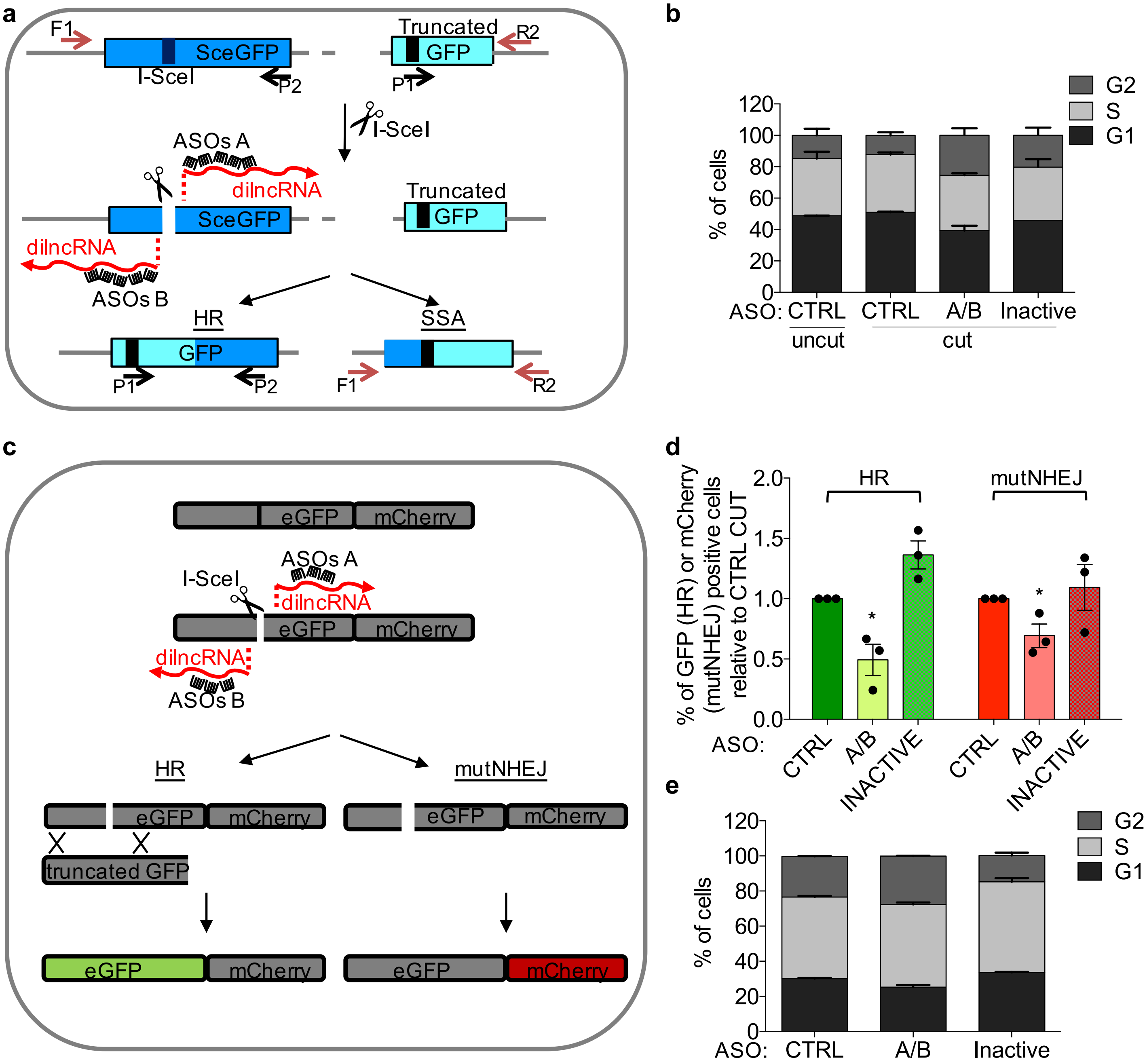

Supplementary Fig. 4: ASOs-mediated dilncRNA inactivation effect on HR and NHEJ

a, Schematic representation of the ASOs used to inhibit dilncRNAs and the primers used to detect homologous recombination (HR) and single strand annealing (SSA) in the DR-GFP system. Upon HR, the correct GFP sequence containing the 5’ portion of the truncated GFP and the 3’ of the sceGFP is generated. The HR product can be amplified by PCR using one primer matching the 5’ part of the truncated GFP (P1) and one primer matching the 3’ part of the sceGFP (P2). Over-resection of the exposed DNA ends results in SSA and generates a 0.8 Kb amplicon when a primer matching a region upstream to the sceGFP (F1) and a primer matching a region downstream of the truncated GFP (R2) are used for PCR. **b**, FACS analysis of the cell-cycle profile of cells treated with control ASO (CTRL), ASOs matching dilncRNAs (A/B), or inactive ASOs A/B. Bar graphs represent mean values from n = 2 independent experiments and error bars represent s.e.m. **c**, Schematic representation of the Traffic Light Reporter (TLR) system. Upon I-SceI-mediated cutting, HR with an externally provided donor results in GFP expression, while mutagenic NHEJ events restoring the frame of mCherry result in mCherry expression. **d**, FACS analysis of the percentage of GFP (HR) and mCherry (mutNHEJ) positive cells upon treatment with control ASO (CTRL), ASOs matching dilncRNAs (A/B), or inactive ASOs A/B. Bar graphs represent mean values from n = 3 independent experiments and error bars represent s.e.m. **e**, FACS analysis of the cell-cycle profile of cells treated with control ASO (CTRL), ASOs matching dilncRNAs (A/B), or inactive ASOs A/B. Bar graphs represent mean values from n = 2 independent experiments. Error bars represent s.e.m. *$P<0.05$ (two-tailed Student’s t test). Source data are provided as a Source Data file.

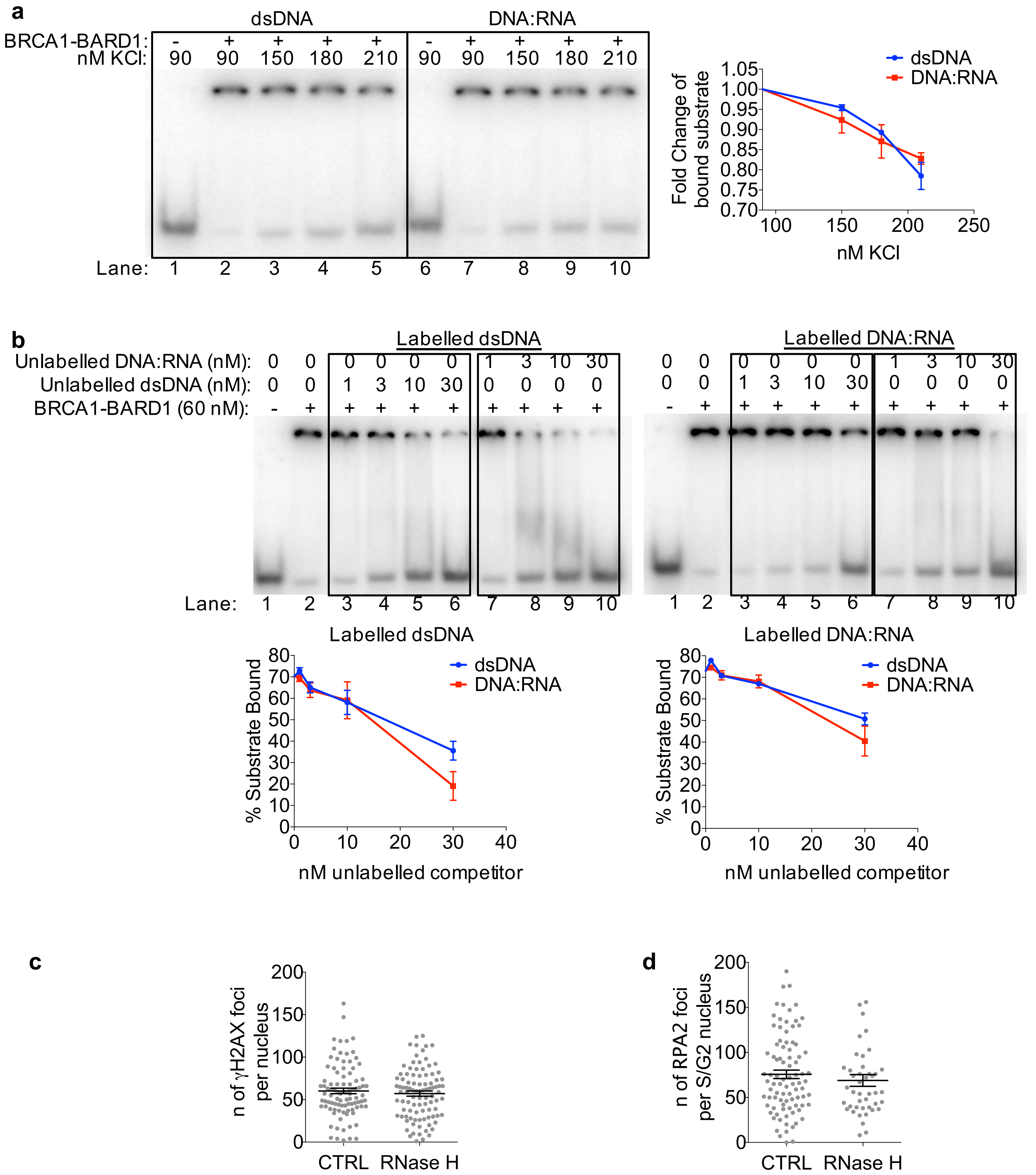

Supplementary Fig. 5: BRCA1-BARD1 EMSA competition assays

a, Immunoblot showing EMSA of BRCA1-BARD1 with either dsDNA or DNA:RNA challenged by increasing salt concentration. The graph shows the percentage of protein-bound substrate at respective concentrations of salt from n = 3 biological replicates. Error bars represent s.e.m. **b**, Immunoblot showing EMSA of BRCA1-BARD1 pre-bound with either dsDNA or DNA:RNA challenged by increasing concentration of unlabelled dsDNA or DNA:RNA hybrids. The graphs show the percentage of protein-bound substrate at respective concentrations of unlabelled competitor from n = 3 biological replicates. Error bars represent s.e.m. **c**, Dot plot shows γH2AX foci in irradiated (2Gy) U2OS cells treated with RNase H prior to fixation. **d**, Dot plot shows RPA2 foci co-stained with cyclin A, as S/G2-phase marker, in irradiated (2Gy) U2OS cells treated with RNase H prior to fixation. In **c** and **d** at least n = 40 cells were counted from 2 independent experiments. Lines represent mean±s.e.m. Source data are provided as a Source Data file.

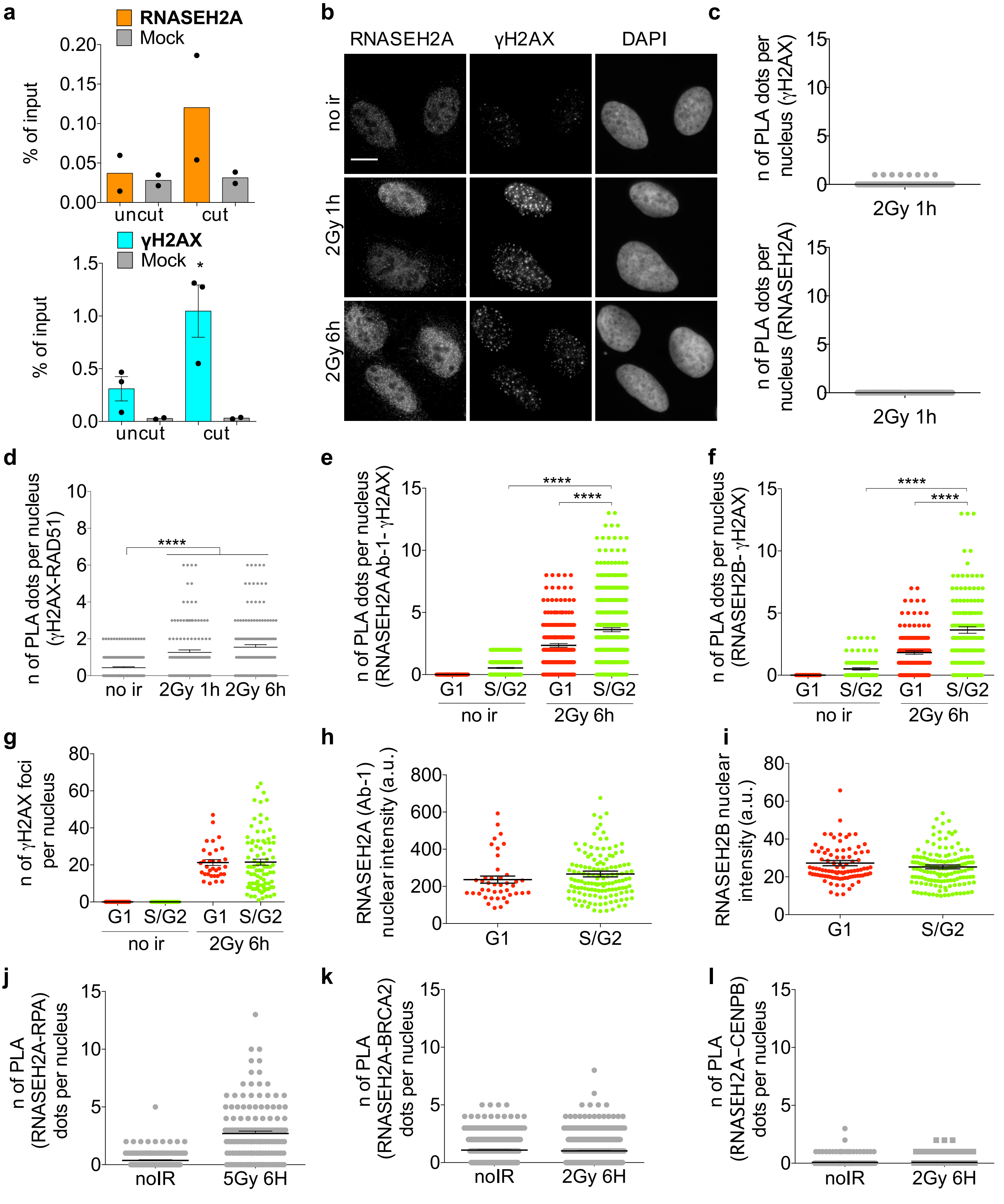

Supplementary Fig. 6: RNASEH2A and DDR proteins PLA and PLA specificity controls

a, ChIP of RNASEH2A (top) and γH2AX (bottom) at the AsiSI cut site in uncut or cut DIvA cells. The bar graphs show the percentage of input in cut cells (6h after AsiSI induction) compared to uncut from 2 (top) or 3 (bottom) biological replicates. Error bars represent s.e.m. **b**, Representative images of RNASEH2A and γH2AX staining in not irradiated (no ir) or irradiated U2OS cells. Scale bar: 10 µm. **c**, Dot plots show the number of signals per nucleus of PLA when only γH2AX or RNASEH2A antibody is used in irradiated (2Gy) U2OS cells. At least n = 600 cells from 3 independent experiments were counted. Lines represent mean±s.e.m. **d**, Dot plot shows the number of signals per nucleus of PLA between γH2AX and RAD51 in irradiated (2Gy) or not irradiated (no ir) U2OS cells. At least n = 120 cells from 3 independent experiments were counted. Lines represent mean±s.e.m. **e,** Dot plots show the number of signals per nucleus of PLA

between γH2AX and RNASEH2A detected with a different antibody (Ab-1) or **f**, γH2AX and RNASEH2B in not irradiated (no ir) or irradiated (2Gy) G1- and S/G2-phase HeLa-FUCCI cells. At least n = 170 cells from 5 independent experiments were counted in **e**. At least n = 80 cells from 2 independent experiments were counted in **f**. Lines represent mean±s.e.m. **g**, Dot plot shows the number of γH2AX foci per nucleus in not irradiated (no ir) or irradiated (2Gy) G1- and S/G2-phase HeLa-FUCCI cells. At least n = 30 cells from 2 independent experiments were counted. Lines represent mean±s.e.m. **h,** Dot plot shows the nuclear intensity of RNASEH2A or **i**, RNASEH2B signals in G1- or S/G2-phase HeLa-FUCCI cells. At least n = 90 cells from 2 independent experiments were counted. Lines represent mean±s.e.m. **j**, Dot plots showing the number of signals per nucleus of PLA between RNASEH2A and RPA from one representative experiment in not irradiated (no ir) or irradiated (5Gy) cells. At least n = 140 cells were counted. Lines represent mean±s.e.m. The experiment was repeated twice with similar results. **k,** Dot plots showing the number of signals per nucleus of PLA between RNASEH2A and BRCA2 or **l**, CENPB, as a negative control in not irradiated (no ir) or irradiated (2Gy) cells. At least n = 300 cells were counted from 2 independent experiments. Lines represent mean±s.e.m. ****$P < 0.0001$ (two-tailed Student's t test). Source data are provided as a Source Data file.

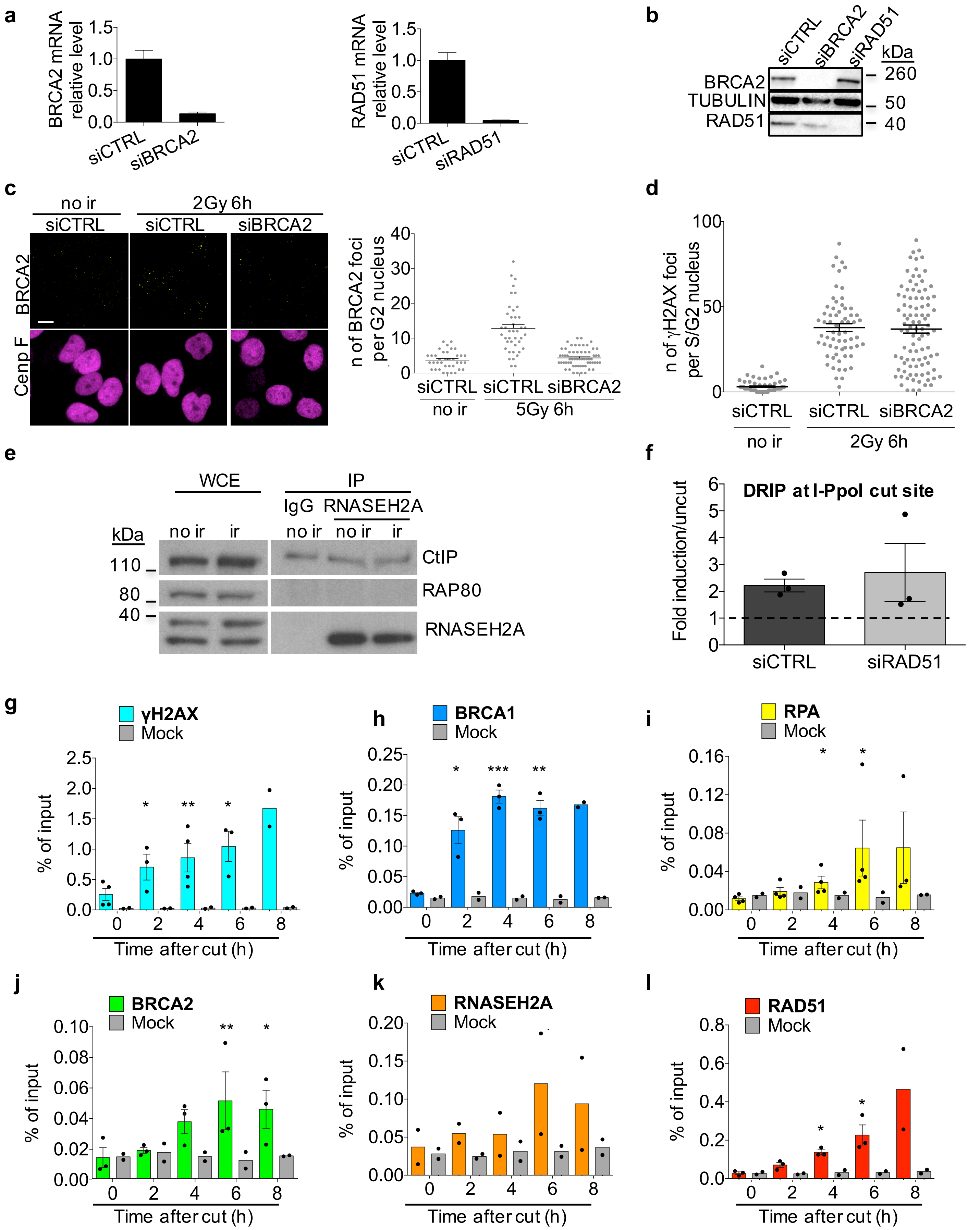

Supplementary Fig. 7: RAD51 knock-down does not affect DNA:RNA hybrid level at DSBs

a, BRCA2 and RAD51 knock-down efficiency monitored by RT-qPCR. One representative experiment is shown. Error bars represent s.d. of the RT-qPCR technical replicates **b**, Representative immunoblot HeLa-FUCCI cells knocked-down for BRCA2 or RAD51. This experiment was repeated two times independently with similar results. **c**, Representative images of BRCA2 immunofluorescence in not irradiated (no ir) or

irradiated (5Gy) HeLa cells knocked-down for BRCA2. Scale bar: 10 µm. Dot plot shows the number of BRCA2 foci per G2 nucleus. One representative experiment is shown. At least n = 40 cells were counted. Lines represent mean±s.e.m. **d**, Dot plot shows the number of γH2AX foci in S/G2-phase HeLa-FUCCI cells knocked-down for BRCA2. At least n = 100 cells were counted from 2 independent experiments. Lines represent mean±s.e.m. **e**, Co-immunoprecipitation of endogenous RNASEH2A from not irradiated (no ir) or irradiated 5Gy (ir) HEK293T cell extract in the presence of benzonase. This experiment was repeated three times independently with similar results. **f**, DRIP-qPCR at 1.5 Kb on the right from the I-PpoI cut site within DAB1 gene in RAD51 knocked-down S/G2-phase-sorted HeLa-FUCCI cells transfected with the I-PpoI nuclease. The bar graph shows the average fold induction of cut samples relative to uncut from n = 3 independent experiments. Error bars represent s.e.m. **g**, γH2AX, **h,** BRCA1, **i**, RPA, **j**, BRCA2, **k**, RNase H2, and **l**, RAD51 percentage of input at the non-genic AsiSI site analysed in Fig. 1d. Error bars represent mean±s.e.m. ($n \geq 2$ biological replicates). *$P < 0.05$, **$P < 0.01$ (two-tailed Student's t test).

Figure 4c

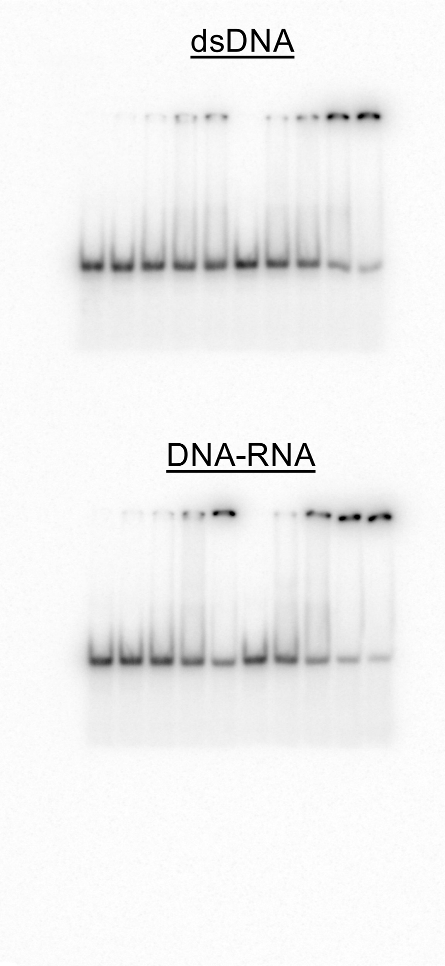

Figure 6c

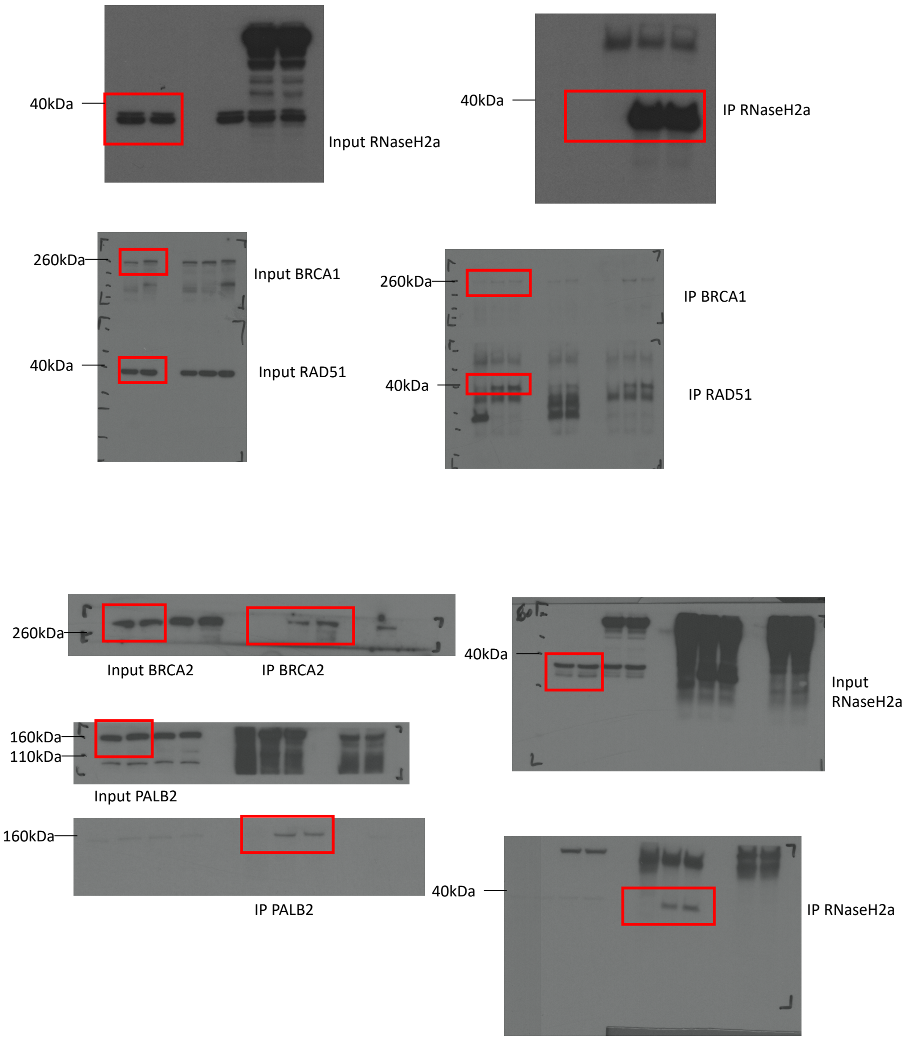

Figure 6d

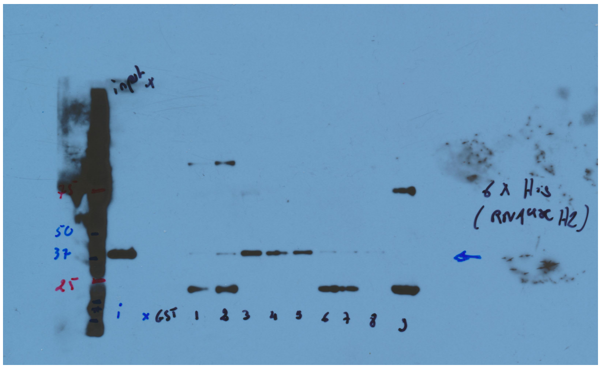

Figure 6e

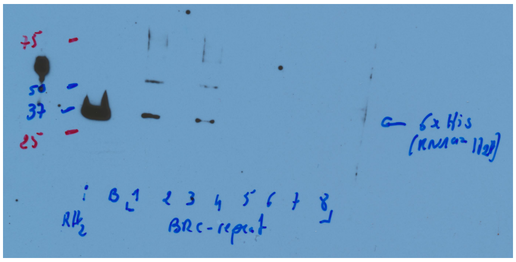

Supplementary Fig. 8: Uncropped scans for main figures blots
