# Supplementary material for: BRCA2 controls DNA:RNA hybrid level at DSBs by mediating RNase H2 recruitment
Source: Nat Commun. 2018 Dec 18;9:5376. doi: 10.1038/s41467-018-07799-2 (PMC6299093; doi:10.1038/s41467-018-07799-2)
Supplement: Supplementary file 2 — Reporting Summary [file 41467_2018_7799_MOESM2_ESM.pdf]

nature research

Corresponding author(s): Giuseppina D'Alessandro, Fabrizio d'Adda di Fagagna

Reporting Summary

Nature Research wishes to improve the reproducibility of the work that we publish. This form provides structure for consistency and transparency in reporting. For further information on Nature Research policies, see Authors & Referees and the Editorial Policy Checklist.

Statistical parameters

When statistical analyses are reported, confirm that the following items are present in the relevant location (e.g. figure legend, table legend, main text, or Methods section).

n/a	Confirmed	
☐	☒	The exact sample size (*n*) for each experimental group/condition, given as a discrete number and unit of measurement
☐	☒	An indication of whether measurements were taken from distinct samples or whether the same sample was measured repeatedly
☐	☒	The statistical test(s) used AND whether they are one- or two-sided<br>*Only common tests should be described solely by name; describe more complex techniques in the Methods section.*
☒	☐	A description of all covariates tested
☒	☐	A description of any assumptions or corrections, such as tests of normality and adjustment for multiple comparisons
☐	☒	A full description of the statistics including central tendency (e.g. means) or other basic estimates (e.g. regression coefficient) AND variation (e.g. standard deviation) or associated estimates of uncertainty (e.g. confidence intervals)
☐	☒	For null hypothesis testing, the test statistic (e.g. *F*, *t*, *r*) with confidence intervals, effect sizes, degrees of freedom and *P* value noted<br>*Give P values as exact values whenever suitable.*
☒	☐	For Bayesian analysis, information on the choice of priors and Markov chain Monte Carlo settings
☒	☐	For hierarchical and complex designs, identification of the appropriate level for tests and full reporting of outcomes
☒	☐	Estimates of effect sizes (e.g. Cohen's *d*, Pearson's *r*), indicating how they were calculated
☐	☒	Clearly defined error bars<br>*State explicitly what error bars represent (e.g. SD, SE, CI)*

Our web collection on statistics for biologists may be useful.

Software and code

Policy information about availability of computer code

Data collection

MetaMorph software was used to acquire widefield images. LCS (Leica Confocal Software) software was used to acquire confocal images. Super-resolution microscopy data were collected and imaged on an electron-multiplying charged coupled device (EMCCD, Andor) using Solis software (Andor).

Data analysis

Quantification of the number of foci per nucleus was performed with the automated image analysis software CellProfiler 2.1.1.
For super-resolution data analysis, each raw image stack was processed for single molecule localization and rendered via rapidSTORM or QuickPALM. Prism 6 software was used to generate graphs and to perform statistical analysis.
For DRIP-seq analysis, raw reads were aligned to the human genome using BWA70, and aligned reads were processed and duplicates were removed using SAMtools. The log2ratio of the fold enrichment of cut vs uncut DRIP-seq samples, was calculated using bamCompare from the suite DeepTools. The distribution profile of DNA:RNA hybrids enrichment at DSBs was calculated using intersectBed tool from BedTools suite.

For manuscripts utilizing custom algorithms or software that are central to the research but not yet described in published literature, software must be made available to editors/reviewers upon request. We strongly encourage code deposition in a community repository (e.g. GitHub). See the Nature Research guidelines for submitting code & software for further information.

Data

Policy information about availability of data

All manuscripts must include a data availability statement. This statement should provide the following information, where applicable:

- Accession codes, unique identifiers, or web links for publicly available datasets
- A list of figures that have associated raw data
- A description of any restrictions on data availability

All relevant data are available from the authors. A reporting summary for this Article is available as a Supplementary Information file. Source data are provided as a Source Data file. DRIP-seq raw FASTQ data were retrieved from the European Nucleotide Archive public repository (https://www.ebi.ac.uk/ena/data/view/PRJEB24001).

Field-specific reporting

Please select the best fit for your research. If you are not sure, read the appropriate sections before making your selection.

☒ Life sciences ☐ Behavioural & social sciences ☐ Ecological, evolutionary & environmental sciences

For a reference copy of the document with all sections, see nature.com/authors/policies/ReportingSummary-flat.pdf

Life sciences study design

All studies must disclose on these points even when the disclosure is negative.

Sample size	Sample size was not pre-determined and it is indicated in the Figure legends.
Data exclusions	In figures 5e, 6b, and Supplementary Fig. 6d,e,f outliers were removed using the ROUT (Robust regression and Outlier removal) method with the Prism 6 software.
Replication	All experiments were repeated at least 3 times, unless stated differently in figure legends. The main observations of the work were reproduced in different human cell lines, in different experimental settings and by different technologies.<br>Sample size and number of independent experiments are clearly stated in the figure legend.
Randomization	No randomization method was used.
Blinding	Investigators were not blinded to group allocation during data collection and analysis. However, quantification of the number of foci per nucleus was performed in an unbiased way with the automated image-analysis software CellProfiler 2.1.1.

Reporting for specific materials, systems and methods

Materials & experimental systems

n/a	Involved in the study
☒	☐ Unique biological materials
☐	☒ Antibodies
☐	☒ Eukaryotic cell lines
☒	☐ Palaeontology
☒	☐ Animals and other organisms
☒	☐ Human research participants

Methods

n/a	Involved in the study
☒	☐ ChIP-seq
☐	☒ Flow cytometry
☒	☐ MRI-based neuroimaging

Antibodies

Antibodies used	See table 4
Validation	Validations are based on the datasheets from the manufactures. BRCA2 antibody validation is shown in Supplementary Fig. 7c.

Eukaryotic cell lines

Policy information about cell lines

Cell line source(s)	HeLa cells (ATCC)<br>U2OS cells (ATCC)<br>AsiSI-ER-U2OS cells (Iacovoni, 2010)<br>HeLa-FUCCI (RIKEN BioResource Center cell bank, Sakaue-Sawano, 2008)<br>TRI-DR-U2OS (Khurana, 2014)<br>HEK293 TLRsce cells (Certo, 2011)
Authentication	HeLa and U2OS cells are authenticated at each batch freezing by STR profiling (StemElite ID System, Promega).
Mycoplasma contamination	All cell lines are negative for mycoplasma.
Commonly misidentified lines (See ICLAC register)	No commonly misidentified cell lines were used.

Flow Cytometry

Plots

Confirm that:

- [ ] The axis labels state the marker and fluorochrome used (e.g. CD4-FITC).
- [ ] The axis scales are clearly visible. Include numbers along axes only for bottom left plot of group (a 'group' is an analysis of identical markers).
- [ ] All plots are contour plots with outliers or pseudocolor plots.
- [ ] A numerical value for number of cells or percentage (with statistics) is provided.

Methodology

Sample preparation	TRI-DR-U2OS and HEK293 TLRsce cells were fixed in 1% formaldehyde for 20 min on ice. Next, cells were washed in PBS with 1% BSA and fixed in 75% ethanol. Fixed cells were washed again in PBS with 1% BSA and stained with propidium iodide (PI) (Sigma-Aldrich, 50 µg/ml) in PBS supplemented with RNase A (Sigma-Aldrich, 250 µg/ml). For cell cycle analysis, cells were directly fixed in 75% ethanol, as described above.<br>HeLa-FUCCI cells were sorted in PBS with 2% FBS and collected in PBS supplemented with RNaseOUT (Thermo Fisher).
Instrument	For DR-GFP experiments, cells were acquired with the FACSCanto II using the FACS Diva Software 6.1.1.<br>For TLR experiments, cells were acquired with the Attune NxT using the Attune NxT Software.<br>HeLa-FUCCI cells were sorted with the MofloAstrios (Beckman Coulter) using the Summit 6.3 software.
Software	For DR-GFP experiments, analysis was performed with the FACS Diva Software 6.1.1.<br>For TLR experiments, analysis was performed with FlowJo V10 software.<br>For HeLa-FUCCI cells, analysis was performed with Summit 6.3 software.
Cell population abundance	The purity of HeLa-FUCCI cells sorted populations was determined by post-sorting.
Gating strategy	For DR-GFP experiments, doublets were removed and living cells selection was based on forward and side scatter. GFP was excited with 488nm laser and acquired with 530/30 BP filter.<br>For TLR experiments, doublets were removed and living cells selection was based on forward and side scatter. GFP was measured using a 488 nm laser for excitation and acquired with BL1channel. mCherry was excited using a 561 nm laser and acquired with YL2 channel. mTagBFP was excited on a 405 nm laser with VL1 channel, and IFP1.4 was measured with a 640 nm laser and acquired with RL1 channel.<br>For HeLa-FUCCI cells, doublets were removed and living cells selection was based on forward and side scatter. Geminin was excited with 488nm laser and acquired with 513/26 BP filter; RFP was excited with 561nm laser acquired with 579/16 BP filter.

- [x] Tick this box to confirm that a figure exemplifying the gating strategy is provided in the Supplementary Information.
