# Supplementary figures and images for: BRCA2 controls DNA:RNA hybrid level at DSBs by mediating RNase H2 recruitment
Source: Nat Commun. 2018 Dec 18;9:5376. doi: 10.1038/s41467-018-07799-2 (PMC6299093; doi:10.1038/s41467-018-07799-2)

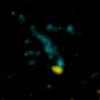

Supplement: Supplementary file 8 — Source Data [file 41467_2018_7799_MOESM8_ESM.zip › TIFF_FIGURES/Figure2b/Fig2B_zoom1.png]

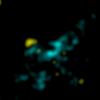

Supplement: Supplementary file 8 — Source Data [file 41467_2018_7799_MOESM8_ESM.zip › TIFF_FIGURES/Figure2b/Fig2B_zoom2.png]

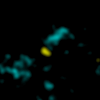

Supplement: Supplementary file 8 — Source Data [file 41467_2018_7799_MOESM8_ESM.zip › TIFF_FIGURES/Figure2b/Fig2B_zoom3.png]

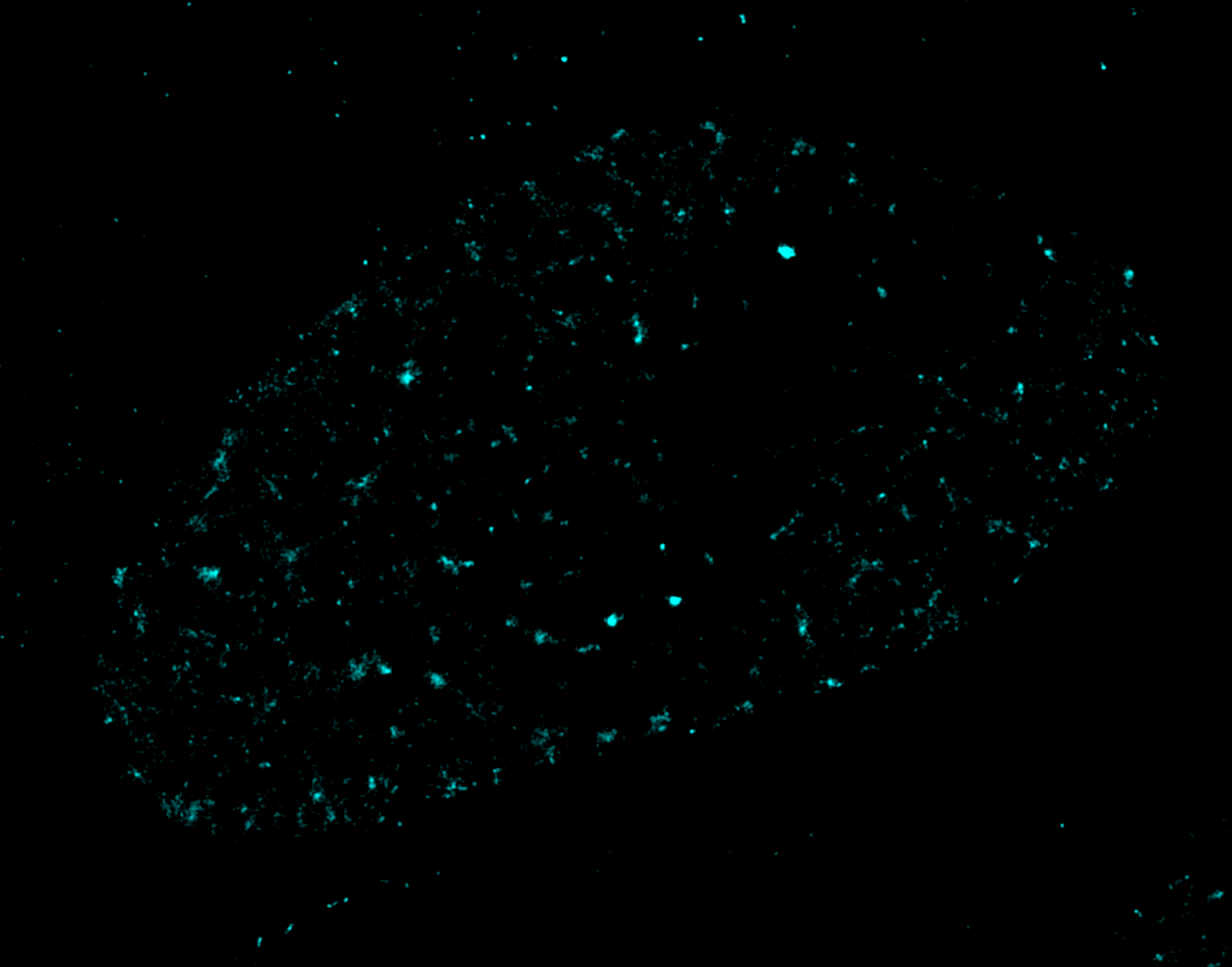

Supplement: Supplementary file 8 — Source Data [file 41467_2018_7799_MOESM8_ESM.zip › TIFF_FIGURES/Figure2b/Fig2Bcyan.png]

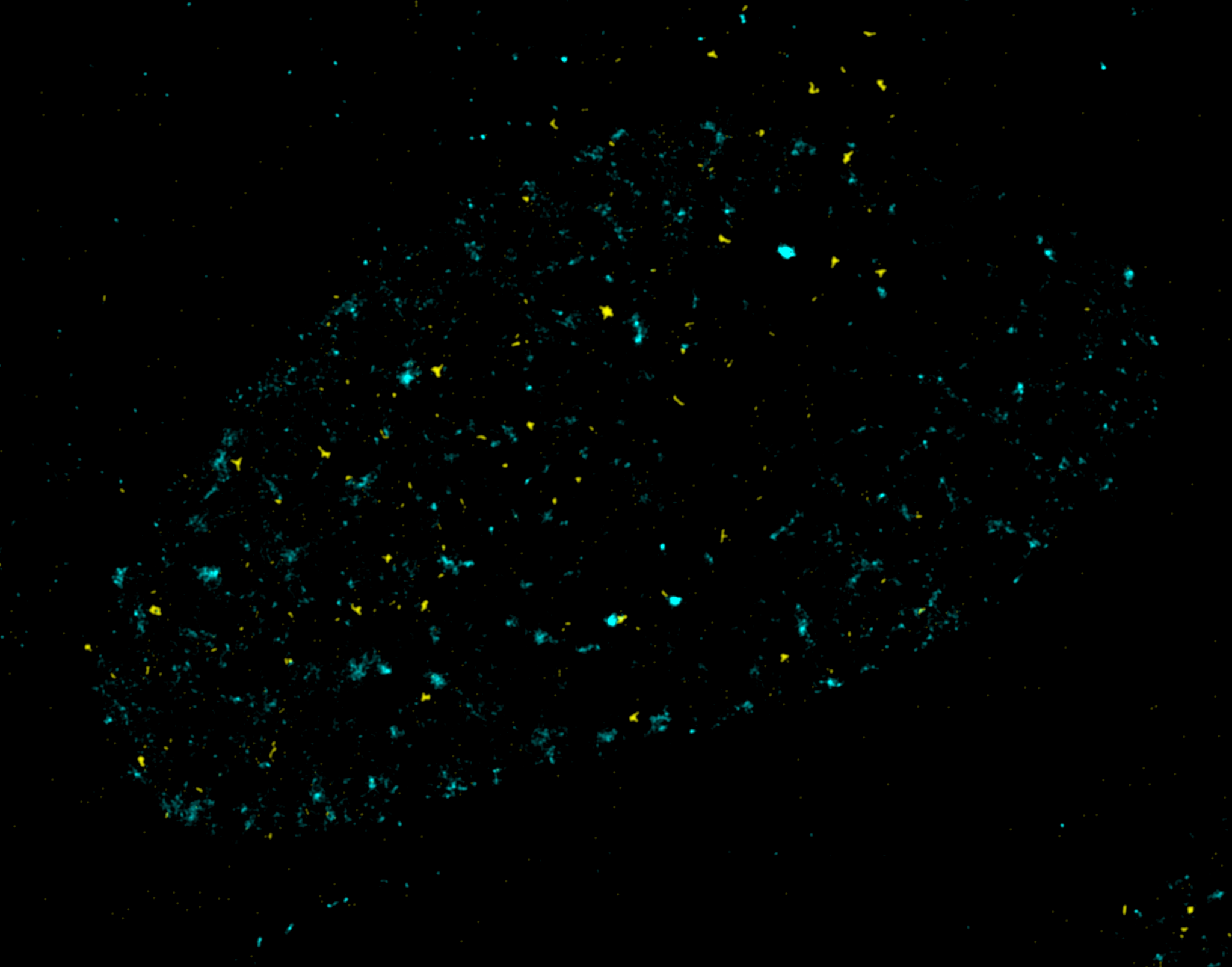

Supplement: Supplementary file 8 — Source Data [file 41467_2018_7799_MOESM8_ESM.zip › TIFF_FIGURES/Figure2b/FIg2Bmerge.png]

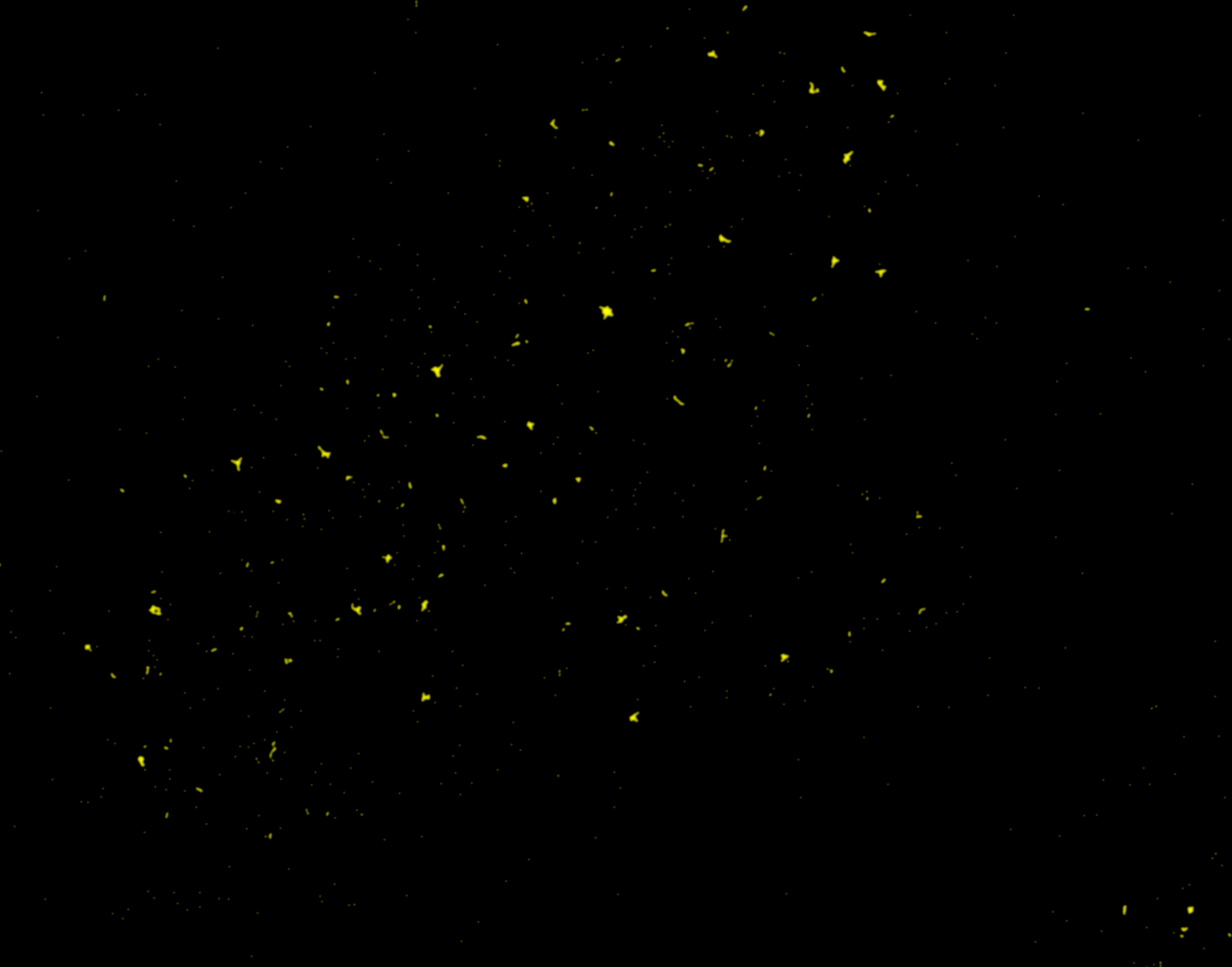

Supplement: Supplementary file 8 — Source Data [file 41467_2018_7799_MOESM8_ESM.zip › TIFF_FIGURES/Figure2b/Fig2Byellow.png]

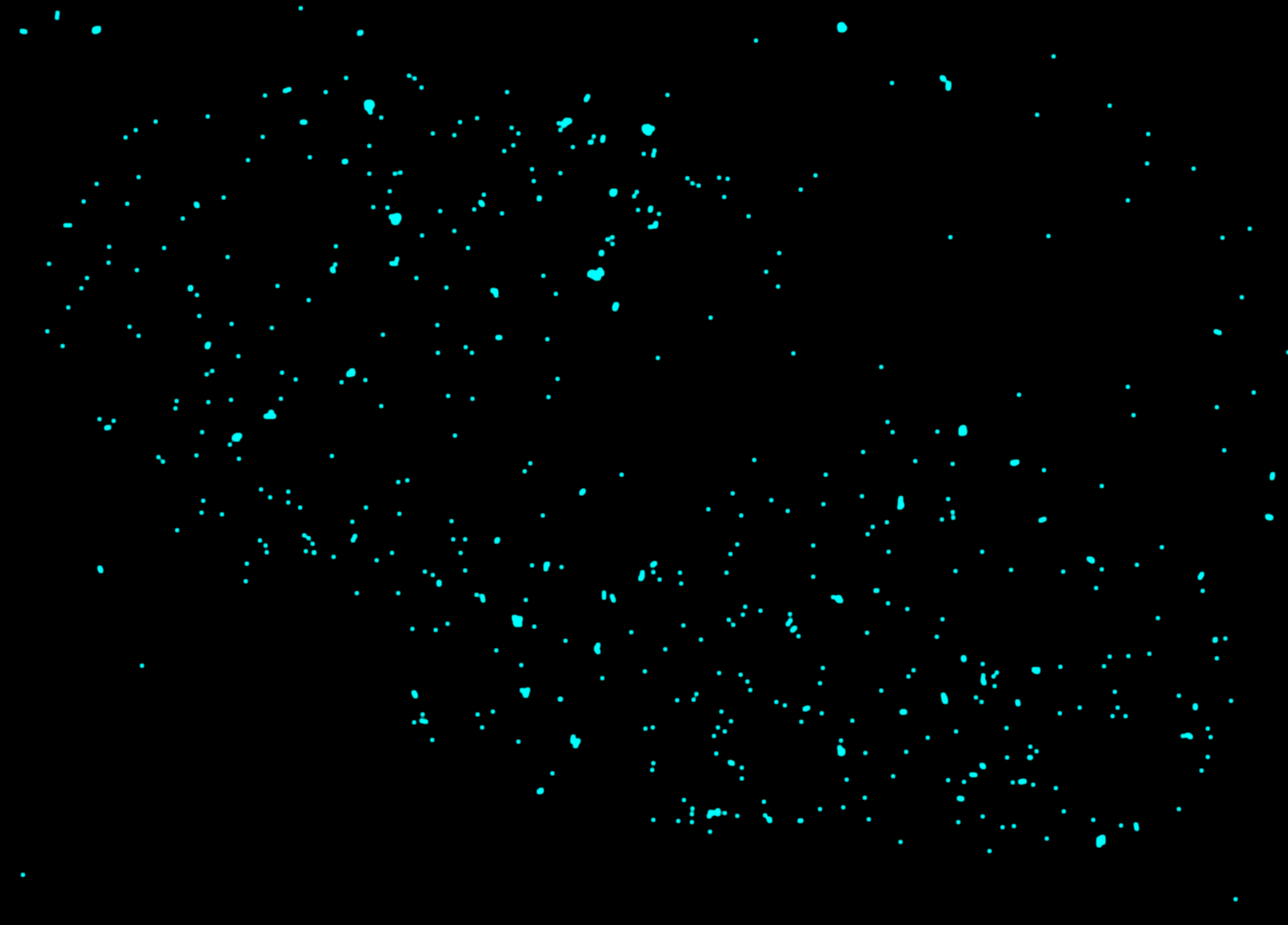

Supplement: Supplementary file 8 — Source Data [file 41467_2018_7799_MOESM8_ESM.zip › TIFF_FIGURES/Figure4a/Fig4a_cyan.png]

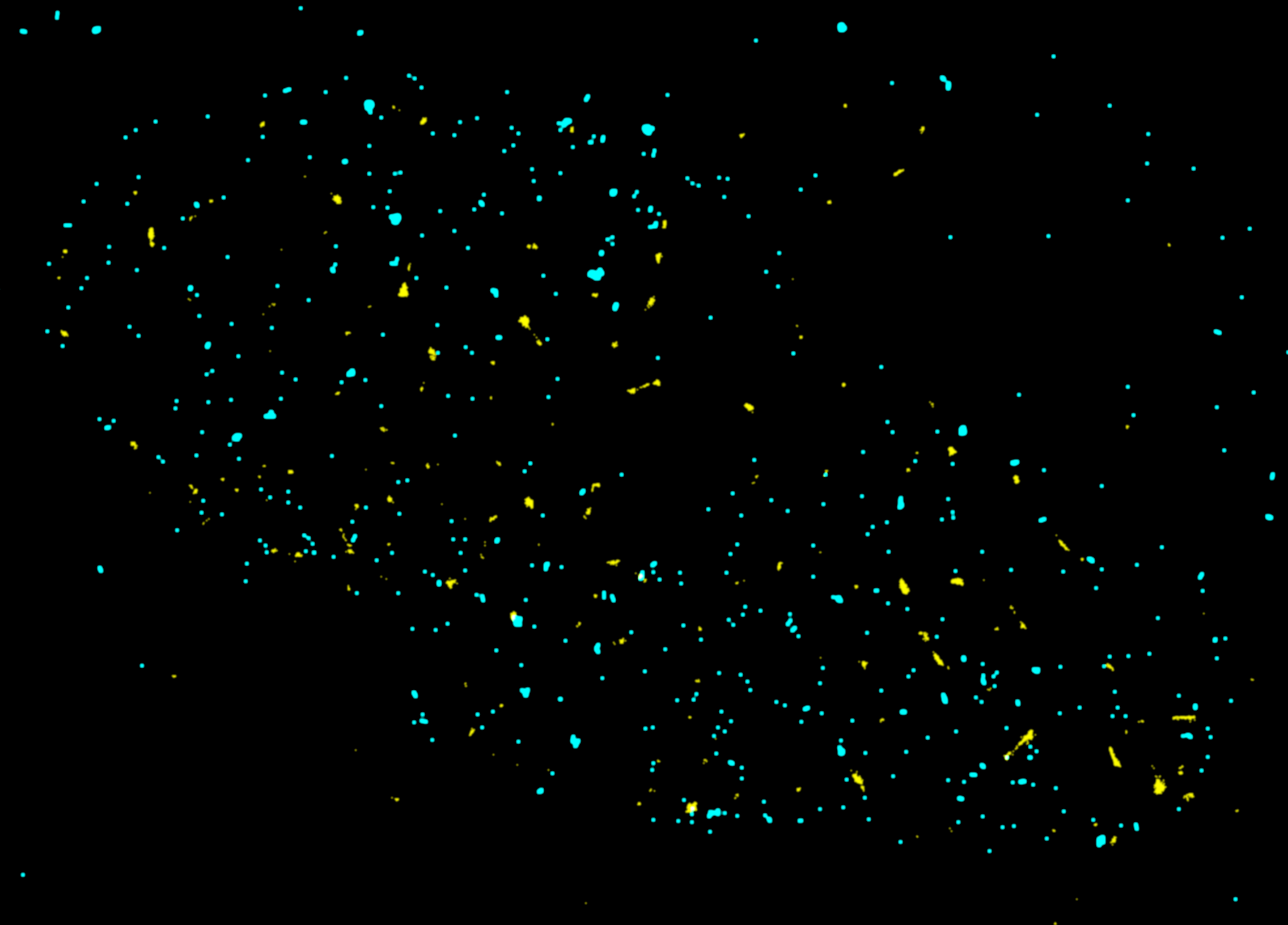

Supplement: Supplementary file 8 — Source Data [file 41467_2018_7799_MOESM8_ESM.zip › TIFF_FIGURES/Figure4a/Fig4a_merge.png]

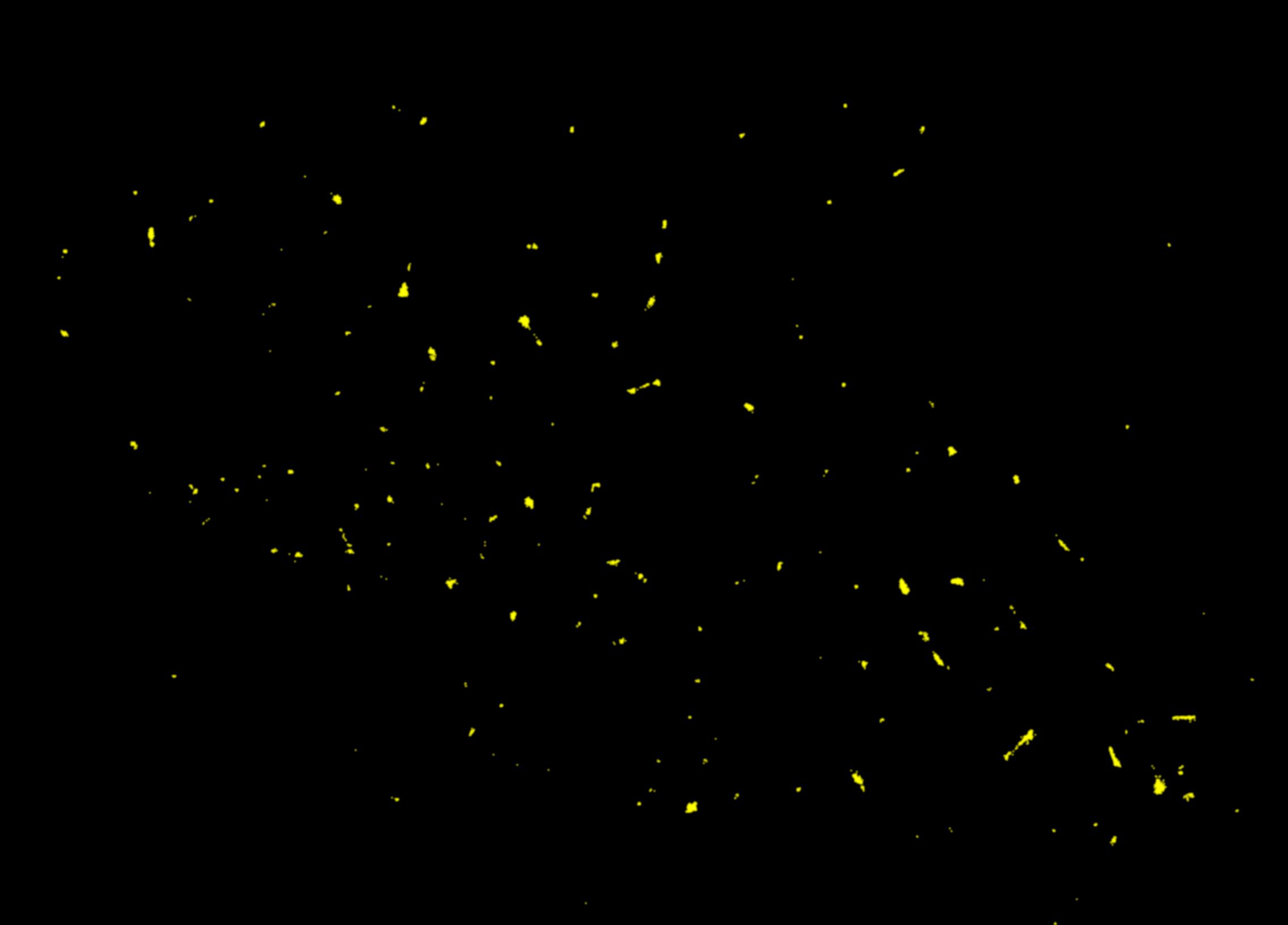

Supplement: Supplementary file 8 — Source Data [file 41467_2018_7799_MOESM8_ESM.zip › TIFF_FIGURES/Figure4a/Fig4a_yellow.png]

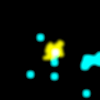

Supplement: Supplementary file 8 — Source Data [file 41467_2018_7799_MOESM8_ESM.zip › TIFF_FIGURES/Figure4a/Fig4a_zoom1.png]

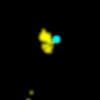

Supplement: Supplementary file 8 — Source Data [file 41467_2018_7799_MOESM8_ESM.zip › TIFF_FIGURES/Figure4a/Fig4a_zoom2.png]

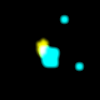

Supplement: Supplementary file 8 — Source Data [file 41467_2018_7799_MOESM8_ESM.zip › TIFF_FIGURES/Figure4a/Fig4a_zoom3.png]

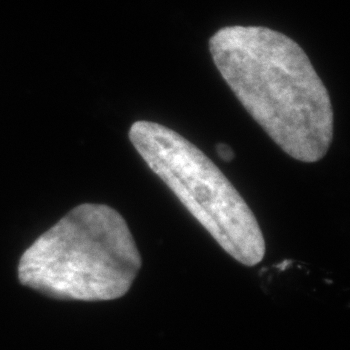

Supplement: Supplementary file 8 — Source Data [file 41467_2018_7799_MOESM8_ESM.zip › TIFF_FIGURES/Figure5b/Fig.5b_1H_composite.tif]

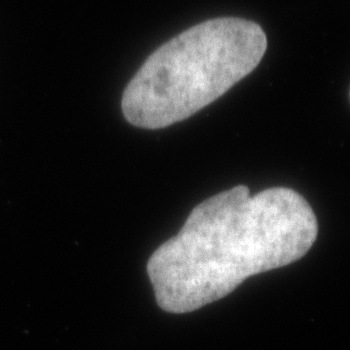

Supplement: Supplementary file 8 — Source Data [file 41467_2018_7799_MOESM8_ESM.zip › TIFF_FIGURES/Figure5b/Fig.5b_6H_composite.tif]

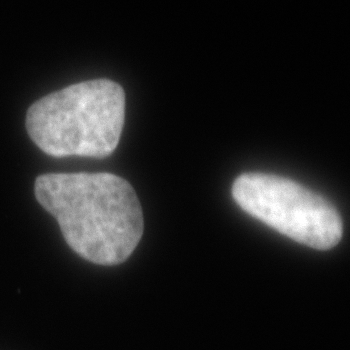

Supplement: Supplementary file 8 — Source Data [file 41467_2018_7799_MOESM8_ESM.zip › TIFF_FIGURES/Figure5b/Fig.5b_noIR_composite.tif]

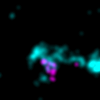

Supplement: Supplementary file 8 — Source Data [file 41467_2018_7799_MOESM8_ESM.zip › TIFF_FIGURES/Figure5f/Fig5f_cyan magenta 2 zi 2 flat.png]

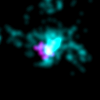

Supplement: Supplementary file 8 — Source Data [file 41467_2018_7799_MOESM8_ESM.zip › TIFF_FIGURES/Figure5f/Fig5f_cyan magenta 2 zi 3 flat.png]

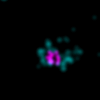

Supplement: Supplementary file 8 — Source Data [file 41467_2018_7799_MOESM8_ESM.zip › TIFF_FIGURES/Figure5f/Fig5f_cyan magenta 2 zi flat.png]

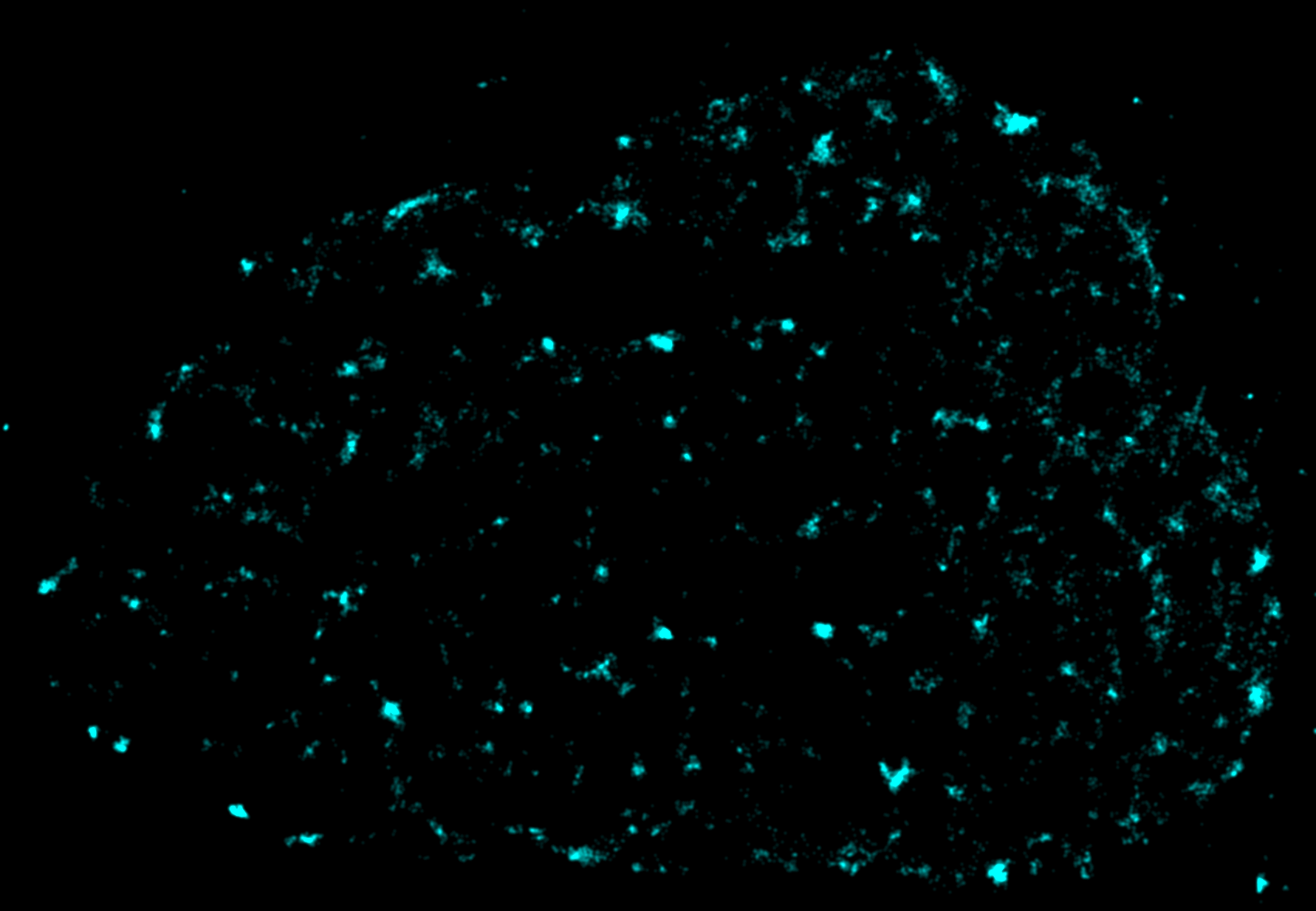

Supplement: Supplementary file 8 — Source Data [file 41467_2018_7799_MOESM8_ESM.zip › TIFF_FIGURES/Figure5f/Fig5fcyan.png]

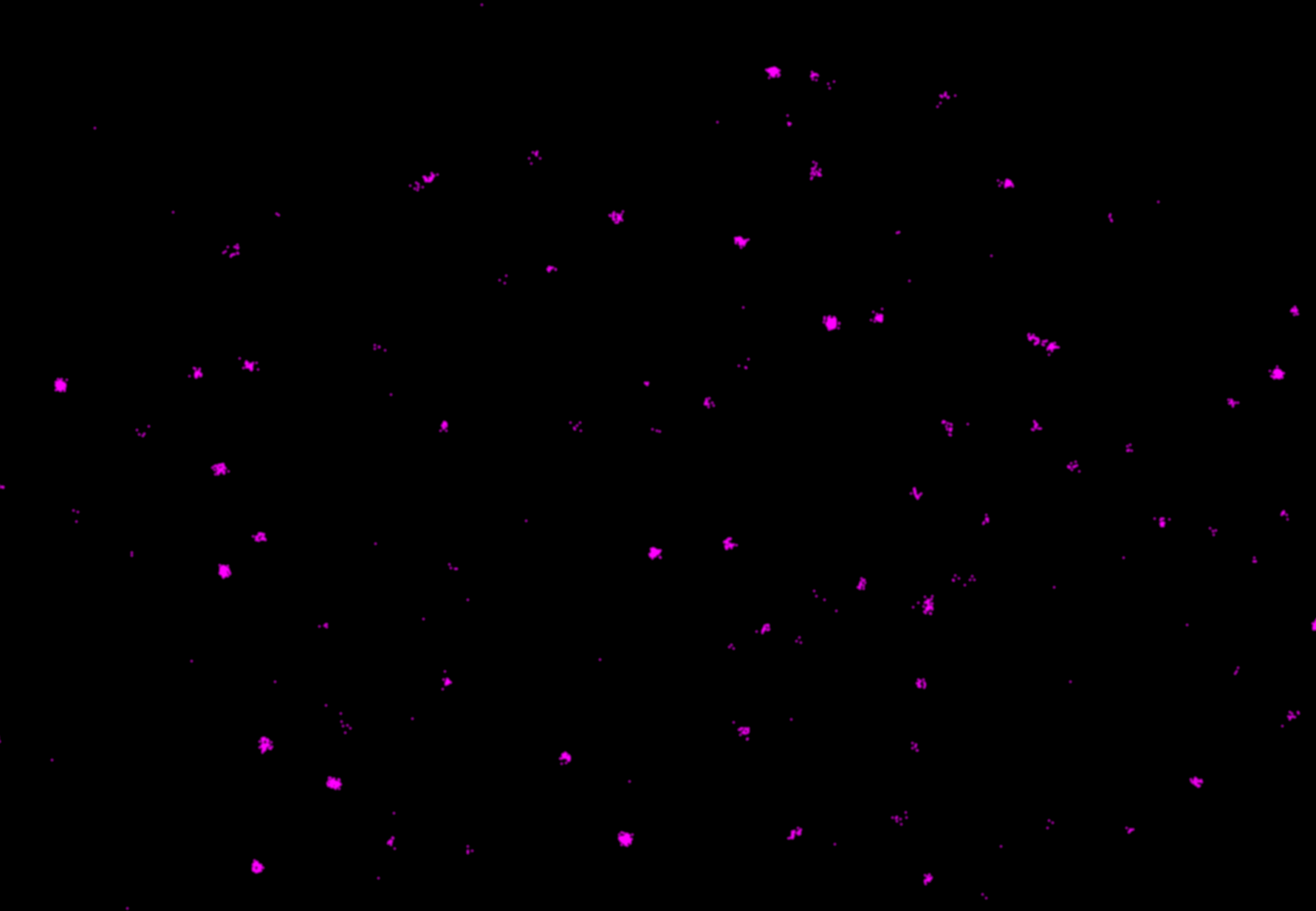

Supplement: Supplementary file 8 — Source Data [file 41467_2018_7799_MOESM8_ESM.zip › TIFF_FIGURES/Figure5f/Fig5fmagenta.png]

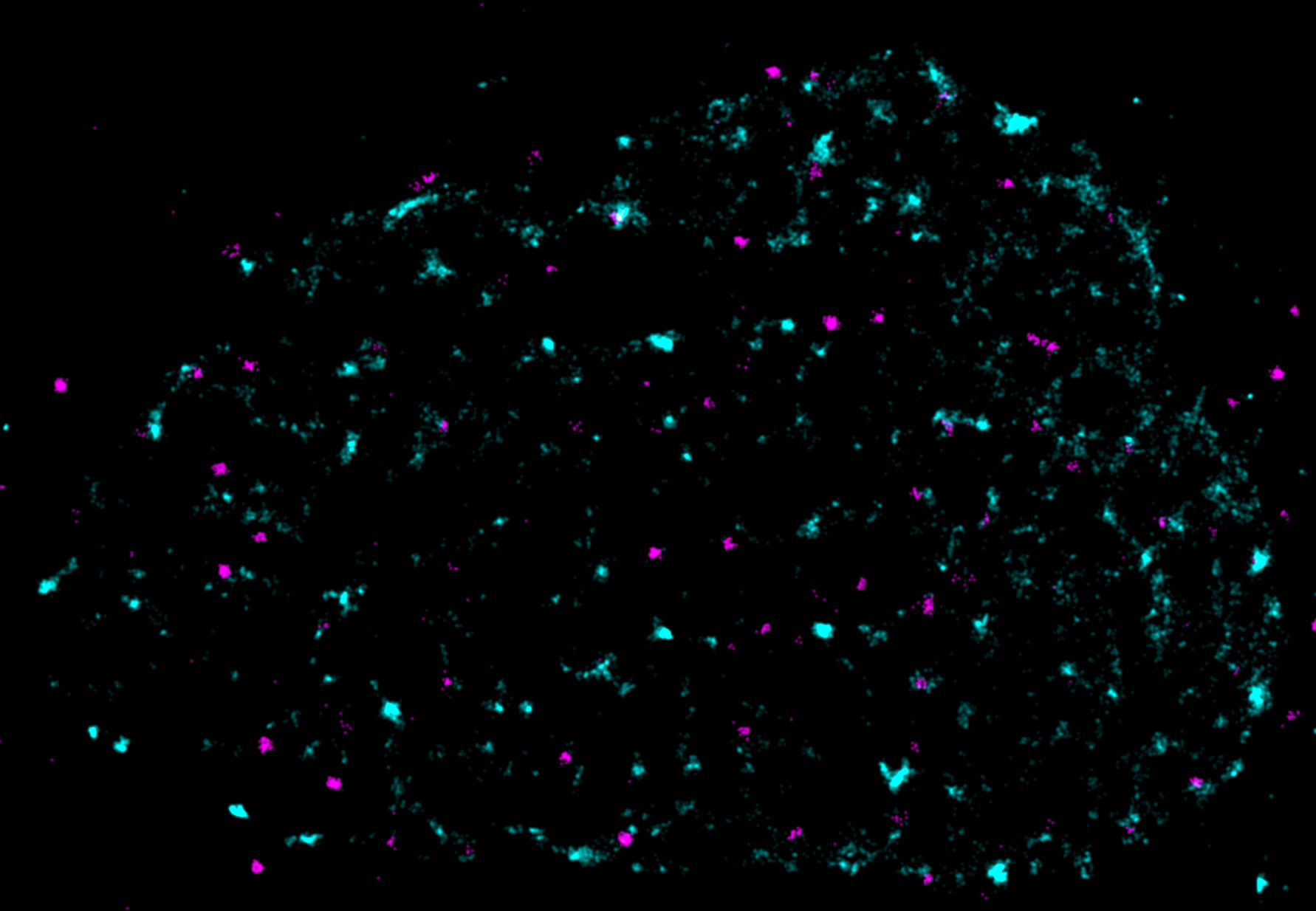

Supplement: Supplementary file 8 — Source Data [file 41467_2018_7799_MOESM8_ESM.zip › TIFF_FIGURES/Figure5f/Fig5fmerge.png]
