# Supplementary material for: BRCA2 controls DNA:RNA hybrid level at DSBs by mediating RNase H2 recruitment
Source: Nat Commun. 2018 Dec 18;9:5376. doi: 10.1038/s41467-018-07799-2 (PMC6299093; doi:10.1038/s41467-018-07799-2)
Supplement: Supplementary file 10 — Description of Additional Supplementary Files [file 41467_2018_7799_MOESM10_ESM.pdf]

Description of Additional Supplementary Files

File Name: Supplementary Data 1

Description: siRNA list

File Name: Supplementary Data 2

Description: ASOs list

File Name: Supplementary Data 3

Description: Primer list

File Name: Supplementary Data 4

Description: Antibody list
